# Layout of ancient Greek papyri through lead-drawn ruling lines revealed by Macro X-Ray Fluorescence Imaging

**DOI:** 10.1038/s41598-023-33242-8

**Published:** 2023-04-21

**Authors:** Francesco P. Romano, Enzo Puglia, Claudia Caliri, Danilo P. Pavone, Michele Alessandrelli, Andrea Busacca, Claudia G. Fatuzzo, Kilian J. Fleischer, Carlo Pernigotti, Zdenek Preisler, Christian Vassallo, Gertjan Verhasselt, Costanza Miliani, Graziano Ranocchia

**Affiliations:** 1 Consiglio Nazionale delle Ricerche, Istituto di Scienze del Patrimonio Culturale (ISPC), Via Biblioteca, 4, 95124 Catania, Italy; 2 Istituto Nazionale di Fisica Nucleare, Laboratori Nazionali del Sud, Via Santa Sofia 62, 95123 Catania, Italy; 3 Dipartimento di Filologia, Letteratura e Linguistica, Università di Pisa, Piazza E. Torricelli, 2, 56126 Pisa, Italy; 4 Consiglio Nazionale delle Ricerche, Istituto per il Lessico Intellettuale Europeo e Storia delle Idee (ILIESI), Via C. Fea, 2, 00161 Rome, Italy; 5 Consiglio Nazionale delle Ricerche, Istituto di Scienze del Patrimonio Culturale (ISPC), Via Card. G. Sanfelice, 8, 80134 Naples, Italy; 6 Dipartimento di Studi Storici, Università degli Studi di Torino, Via Sant’Ottavio, 20, 10124 Turin, Italy; 7 Dipartimento di Scienze Storiche, Geografiche e dell’antichità, Università di Padova, Via del Vescovado, 30, 35141 Padova, Italy

**Keywords:** Applied physics, Archaeology

## Abstract

The use of lead-drawn ruling lines by ancient scribes for the layout of Greek papyrus rolls was known to us only from classical authors and was postulated by a few scholars in modern times. In situ application of noninvasive Macro X-Ray Fluorescence Imaging Spectroscopy (MA-XRF) to unrolled papyri from Herculaneum, dating from about 200 BC to the 1st century AD, has provided the first direct evidence of such practice in ancient book production. The key experimental proof of periodic lines drawn in lead was gathered by a highly sensitive MA-XRF mobile instrument, which allowed detection of ultra-low trace residues of metals with detection limits that rival synchrotron light instruments. Elemental distribution maps of Pb have revealed three different systems of textual layout in ancient papyrus rolls and have resolved the dispute around so-called Maas’ Law, by delivering experimental proof that slanted text columns were a deliberate aesthetic choice of scribes.

## Introduction

It is well-established that Greek and Latin literary works were written on papyrus rolls organized in text columns of regular size. Columns were separated by blank spaces, or intercolumns, of equal width. The first and the last line of each column were carefully written at the same height of the roll, so as to leave a regular blank space both at the top (upper margin) and at the bottom (lower margin) of the roll, with the latter usually being slightly larger than the former^1,2^. The scribe’s goal was to realize the tidiest and most pleasant book possible through the accurate and regular alternation of written and non-written parts. In general, the width of the non-written parts (intercolumns, margins and *agrapha*, i.e. blank spaces left by the scribe at the beginning and the end of the roll) was an indicator of the manuscript’s quality, with broader blank areas corresponding to a more luxurious book. All this required that scribes were able to organize their work before they started transcribing the text, by preliminarily discriminating the written space from the space to be left blank. Up to now we knew about methods and instruments used to this purpose only from a few classical authors such as Catullus, Pliny the Elder and some epigrammatists^3^. The Greek epigrammatist Phanias (2nd-1st century BC) mentions, among the scribe’s tools, a ruler and a “piece of lead used as a marker” (*Palatine Anthology* 6. 295). In the 1st century BC, the Roman poet Catullus criticizes Suffenus’ awful verses for being written on luxurious books in which all parts are “squared through lead” (*carm.* 22). Philip of Thessalonika (AD 30/40) includes among the scribe’s equipment, along with a ruler, a leaden disk “which marks the side of the columns” (*Palatine Anthology* 6. 62). Shortly afterwards, the encyclopedist Pliny the Elder attests that one can trace lines with a piece of lead (certainly also on papyrus), resulting in dirtying one's hands by the material that comes off (*NH* 33. 60). This information is confirmed by another six epigrams belonging to various authors from the *Palatine Anthology* (6. 63–68), which describe the tools consecrated to a deity by an old scribe who is retiring from his job. In all these cases, the scribe’s equipment regularly includes, among other tools, both a ruler and a leaden disk. In conclusion, it can be inferred from these testimonies, ranging from the 2nd century BC to the 6th century AD, that the scribe’s tools remained the same for many centuries and, in particular, that ancient scribes used leaden disks with rounded and smoothened borders to design the layout of the papyrus surface. By keeping the disk between the thumb and the forefinger, they drew it along the rightly positioned ruler, so that the metal, being barely pressured so as not to damage the papyrus fibers, would leave a blunt grey line on the papyrus surface, just noticeable enough for the scribe’s trained eye. According to the *Palatine Anthology* (6. 62), the ruling lines drawn by scribes marked the top and the bottom as well as the left and the right borders of each text column; this implies at the same time a delimitation of the corresponding intercolumns. It stands to reason that every scribe had his own working method, either inherited from his own master or developed by himself over the years. As a consequence, scribes might not have followed one and the same method and, occasionally, they might even have felt free to design the layout of the roll relying only on their own training and accuracy or simply following the horizontally running papyrus fibers as guidelines.

The first modern scholars who alluded to the use of rulers and leaden disks for the layout of manuscripts were, in 1629, Claude de Saumaise and, in 1756, Christian Schwarz^4,5^. In 1805, Johann Beckmann documented that some medieval manuscripts from the 11th and 12th century exhibited lead-drawn ruling lines^6^. Between the 19th and the 20th century, Viktor Gardthausen maintained that, in most cases, only two consecutive vertical lines were drawn by scribes to mark the left border of each column and that, in some cases, horizontal lines were additionally traced to mark single lines within a column. Gardthausen specified that remnants of the latter are sometimes still visible in Herculaneum papyri and postulated that they either disappeared over time or were deleted by scribes after redacting the text^7^. To our knowledge, these ruling lines have never been ascertained in Herculaneum papyri, nor has Gardthausen’s claim been further investigated or verified. Later, Eric Turner hypothesized that ruling lines were drawn on Greek papyri by some material such as lead, which is no longer detectable^8^. However, just like Gardthausen, he was unable to assert whether these traces disappeared on their own after some time or were instead intentionally deleted by scribes. Turner mentions Gardthausen’s testimony to the observation of faint leaden ruling lines in Herculaneum papyri, but at the same time refers back to Guglielmo Cavallo’s silence on them^9^. At any rate, he excludes with all certainty their presence in Greco-Egyptian literary papyri. More recently, Mario Capasso has confirmed that leaden disks were probably used to mark the space devoted to each column^2^. The study presented here offers the first experimental evidence of what we could previously infer only from literary sources about the layout of papyrus bookrolls by Greek scribes by means of lead-drawn ruling lines. Another important result is to be added to this picture. It is a common observation that the text in ancient papyri is organized in skewed columns, with each line starting slightly to the left of the preceding one. Such slanting of the text columns can be more or less pronounced, with an angle of up to 5 degrees, and is known in papyrology as Maas’ Law (named after the classicist Paul Maas)^1^. Up to now, no consensus has been reached among scholars as to whether this was a deliberate and aestheticizing practice or simply the unintentional result of the scribe’s inaccuracy. This research delivers the first experimental proof that slanted columns were a deliberate aesthetic choice of scribes, thus resolving this long-standing controversy.

## Results

In the last decade many scientific studies have been performed to better elucidate the materials of ancient written texts. The investigation of degraded and brittle materials, such as the carbonized Herculaneum papyri discussed here, requires the use of nondestructive and mobile analytical methods since transport and manipulation represent a great risk of damage. MA-XRF imaging is a noninvasive analytical technique developed within synchrotrons facilities^10^ and nowadays consolidated even in mobile instrumentation^11–16^. It has proved extremely useful in the study of paintings^17–22^ and, more recently, of other ancient materials including inks and metal point drawings^23–26^. Notably, the XRF investigation of the most ancient texts written with carbonaceous inks were typically performed within synchrotron facilities, and have focused on the detection of low traces of metals in macroscopic ink marks on small fragments ^27–31^. The current experiment was performed in situ, in the premises of the Officina dei Papiri Ercolanesi of the National Library “Vittorio Emanuele III” in Naples, by using a highly sensitive MA-XRF scanning instrument suited for the detection of ultra-low traces of residual metals across the full macroscopic area of unrolled carbonized papyri.

In particular, Macro-X-Ray Fluorescence Imaging (MA-XRF) was applied to papyrus fragments belonging to six different Herculaneum rolls, dating from the second century BC to the first century AD. They were mechanically unrolled between the 18th and the 19th century and contain works by Epicurus, possibly Demetrius Laco and Philodemus, with the notable case of Philodemus’ *Index Academicorum*, which is transmitted through both a draft in *PHerc*. 1691/1021 and a later final copy in *PHerc.* 164. The different characteristics of the analyzed rolls are summed up below in Methods, *Samples description*.

The results obtained were made possible thanks to the use of the advanced MA-XRF instrument based on real-time technology and providing on-the-fly imaging capabilities, previously developed by the authors^15^. Recently, this instrument underwent a major upgrade, with the integration of new mechatronics and a 3D multi-detector array composed of 6 SDDs operated in parallel and arranged in a compact hodoscope geometry (see Fig. S1). This development dramatically increased the throughput of fluorescence radiation while minimizing the dead time, with the consequent steep improvement of the overall sensitivity. A figure of merit of the upgraded system is given in Fig. S2, also in comparison with a typical single-detector set-up available in a synchrotron facility^32^. This has allowed us to map for the first time in situ even very small traces of metallic elements in this kind of sample—a possibility usually offered only by advanced techniques available in large-scale infrastructures^30^. The lead-drawn markings have been unveiled by distribution maps of the signal coming from the fluorescence M and L lines of lead. Higher concentration of lead has also been found along the borders of some lacunae, possibly caused by the migration of soluble compounds of lead present as contaminants. This last aspect deserves further investigation which is beyond the scope of the present study.

**Figure 1:**
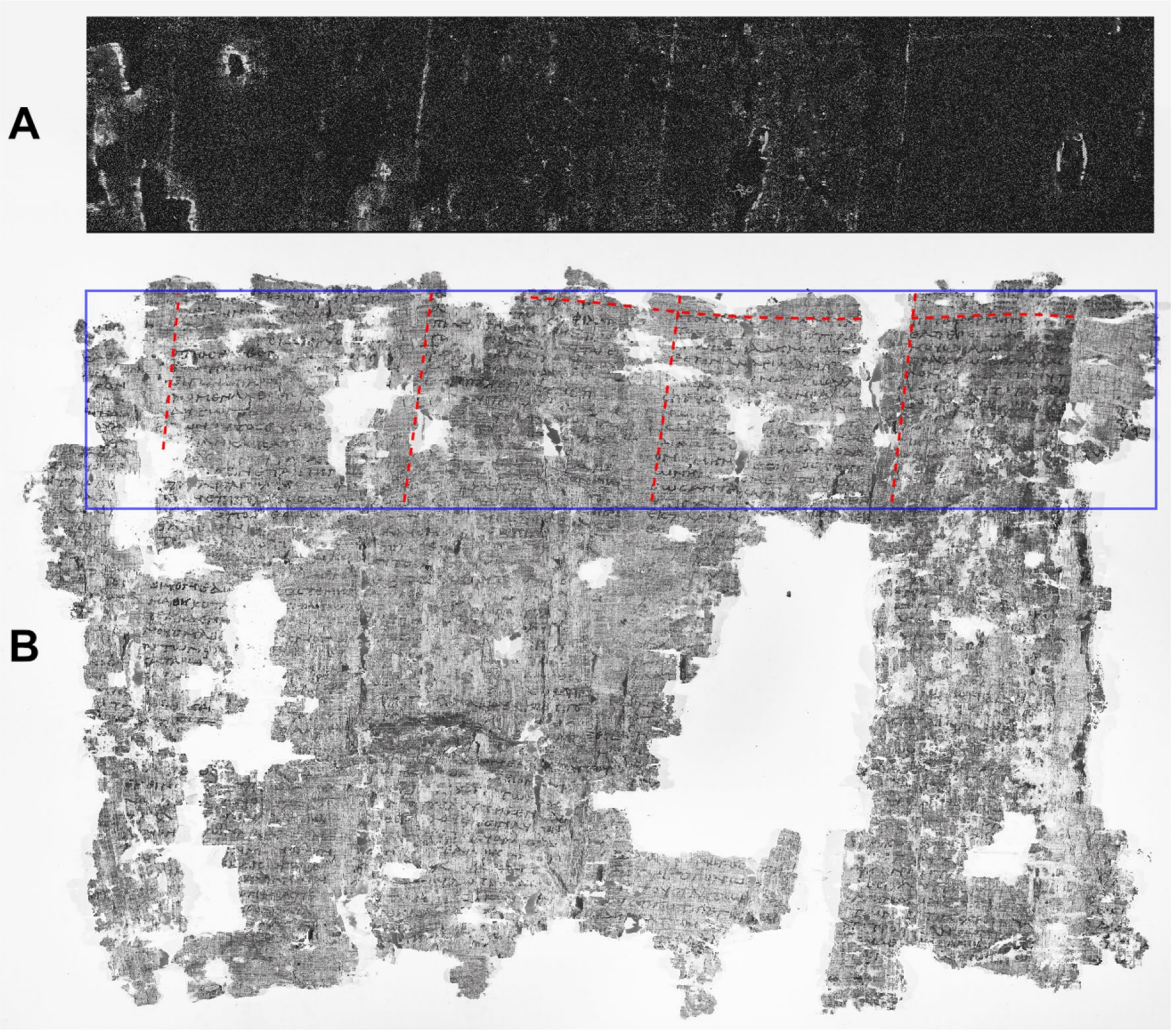
*PHerc.* 1021, ‘cornice’ 1. Pb distribution map obtained by MA-XRF scanning. By permission of the Ministero della Cultura (photo credit: Biblioteca Nazionale “Vittorio Emanuele III,” Napoli—Consiglio Nazionale delle Ricerche, Istituto di Scienze del Patrimonio Culturale) (**A**) and NIR image at 1000 nm. Red lines mark the borders of each intercolumnar space (column + intercolumn); the violet rectangle marks the sample area imaged by us. By permission of the Ministero della Cultura (photo credit: Biblioteca Nazionale “Vittorio Emanuele III,” Napoli—Consiglio Nazionale delle Ricerche, Istituto di Scienze del Patrimonio Culturale) (**B**).

**Figure 2:**
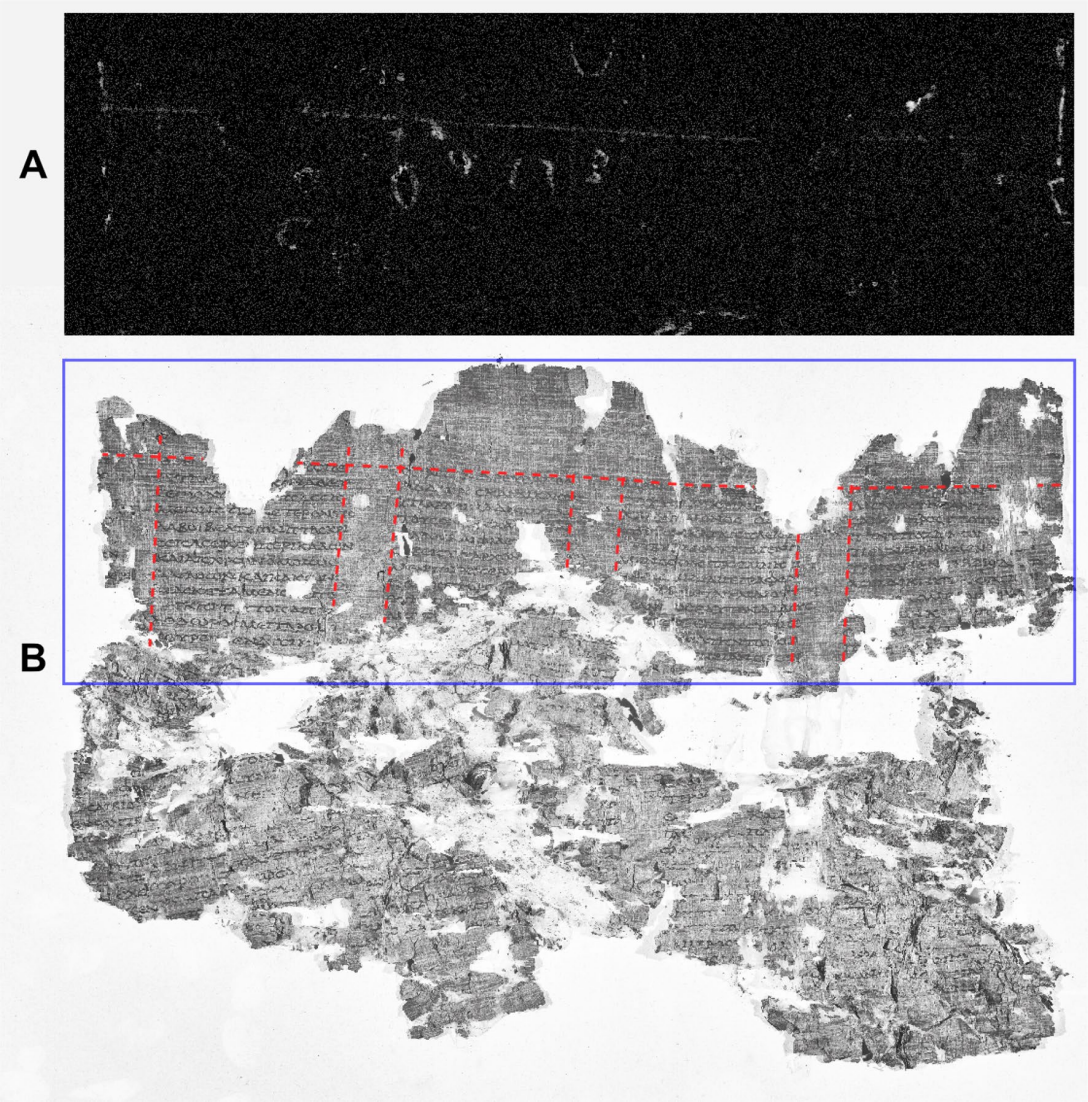
*PHerc.* 1018, ‘cornice’ 1. Pb distribution map obtained by MA-XRF scanning. By permission of the Ministero della Cultura (photo credit: Biblioteca Nazionale “Vittorio Emanuele III,” Napoli—Consiglio Nazionale delle Ricerche, Istituto di Scienze del Patrimonio Culturale) (**A**) and NIR image at 1000 nm. Red lines mark the borders of each column and intercolumn; the violet rectangle marks the sample area imaged by us. By permission of the Ministero della Cultura (photo credit: Biblioteca Nazionale “Vittorio Emanuele III,” Napoli—Consiglio Nazionale delle Ricerche, Istituto di Scienze del Patrimonio Culturale) (**B**).

**Figure 3:**
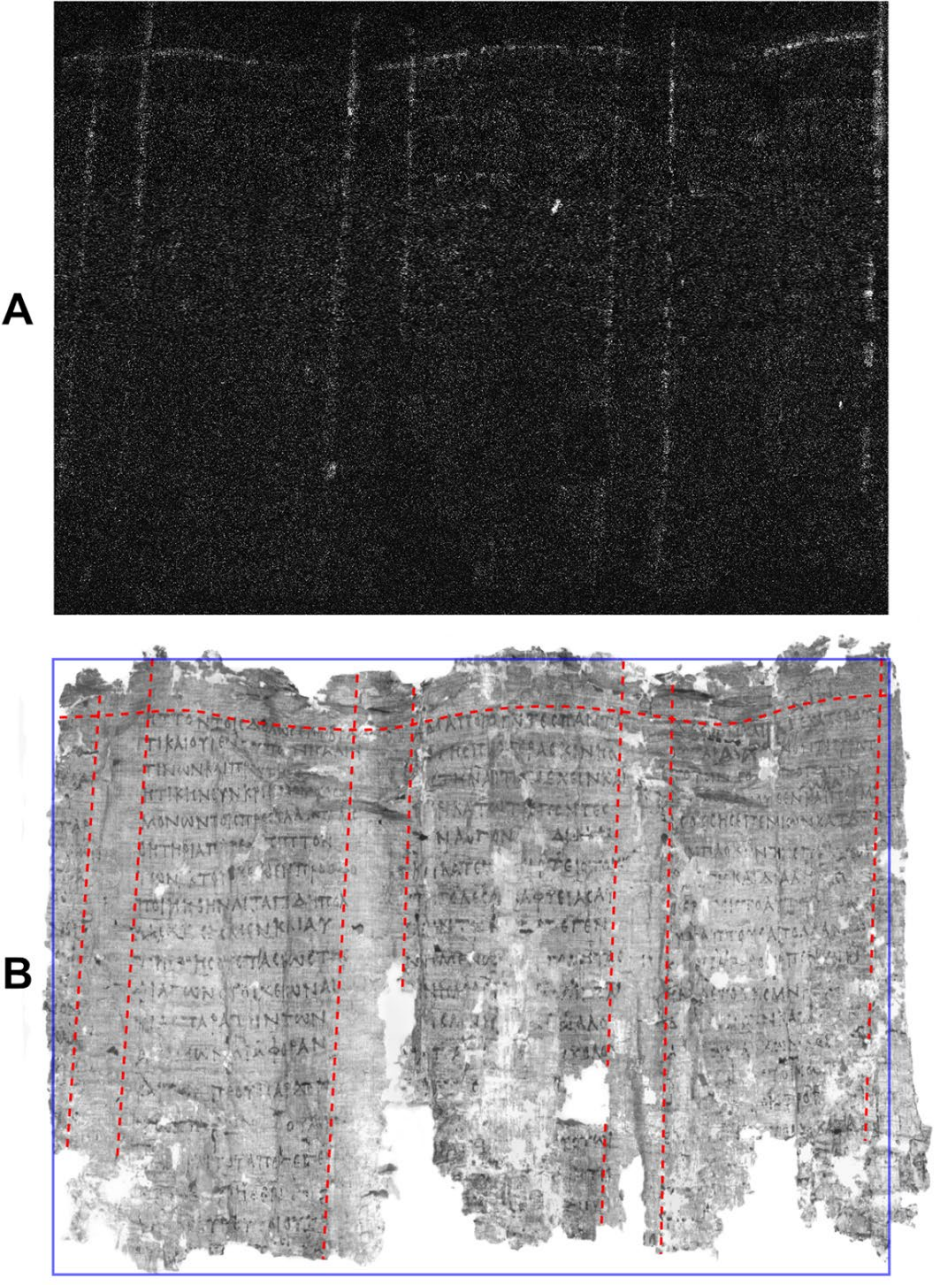
*PHerc.* 1420, ‘cornice’ 2. Pb distribution map obtained by MA-XRF scanning. By permission of the Ministero della Cultura (photo credit: Biblioteca Nazionale “Vittorio Emanuele III,” Napoli—Consiglio Nazionale delle Ricerche, Istituto di Scienze del Patrimonio Culturale) (**A**) and NIR image at 950 nm. Red lines mark the borders of each column and intercolumn; the violet rectangle marks the sample area imaged by us. By permission of the Ministero della Cultura (photo credit: Biblioteca Nazionale “Vittorio Emanuele III,” Napoli—Brigham Young University, Provo) (**B**).

**Figure 4:**
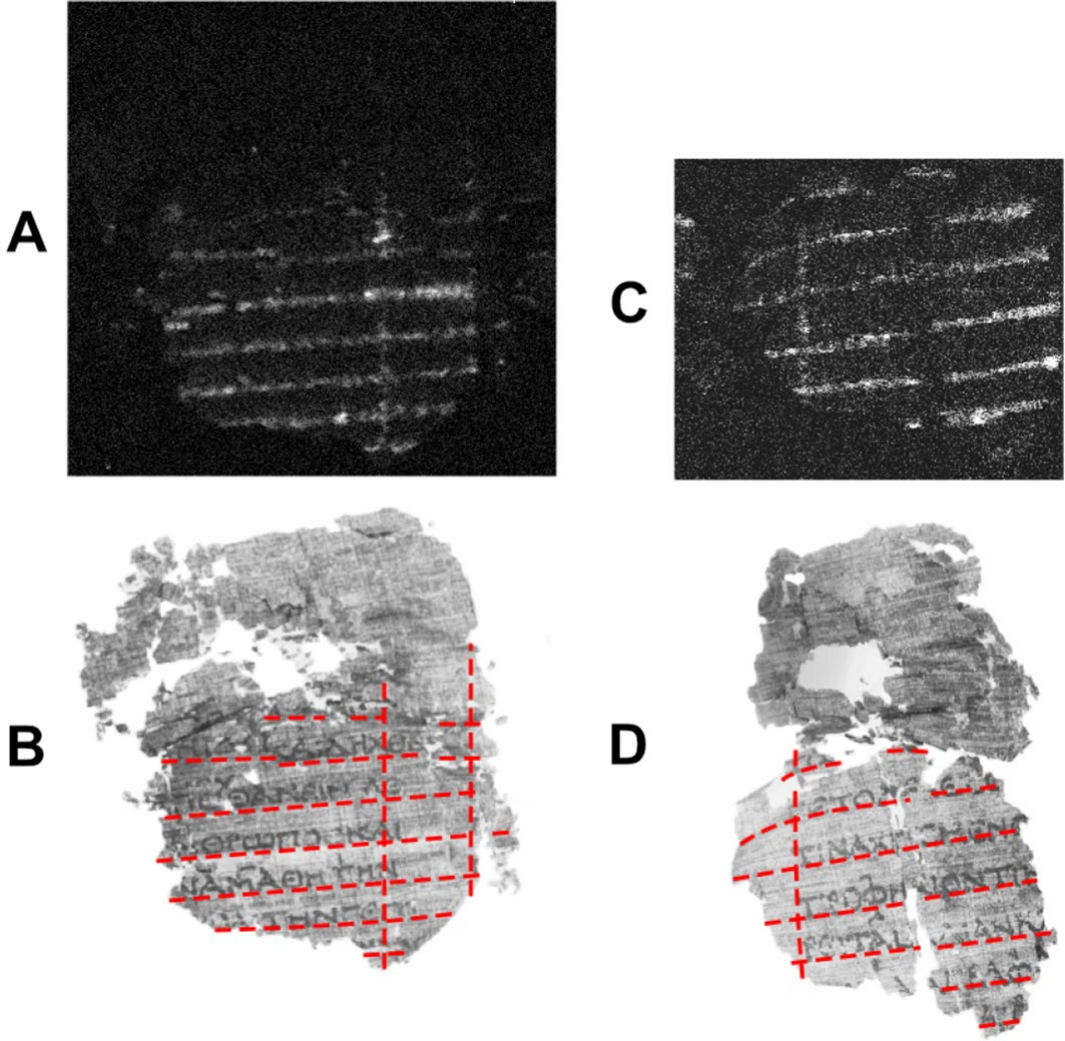
*PHerc.* 164, ‘cornice’ 2, ‘pezzo’ 1. Pb distribution map obtained by MA-XRF scanning. By permission of the Ministero della Cultura (photo credit: Biblioteca Nazionale “Vittorio Emanuele III,” Napoli—Consiglio Nazionale delle Ricerche, Istituto di Scienze del Patrimonio Culturale) (**A,C**) and NIR image at 950 nm. Red lines mark the borders of each column, intercolumn and line. By permission of the Ministero della Cultura (photo credit: Biblioteca Nazionale “Vittorio Emanuele III,” Napoli—Consiglio Nazionale delle Ricerche, Brigham Young University, Provo) (**B,D**).

Due to the large number of specimens and the limited time available for the measurements, only a few selected samples were fully investigated, while limited areas of interest were chosen on the rests of samples. The MA-XRF elemental maps have been directly compared with NIR images of the same papyrus fragment, in order to show the exact correspondence of the lead-drawn lines with the borders between written and blank spaces (see Figs. 1, 2, 3, 4). The NIR images were either collected by us or by a team from Brigham Young University^33^, as indicated in the text. Through these comparisons, three different types of layouts were identified and classified based on the delimitations found.

### Delimitation of intercolumnar spaces

The lead distribution map obtained by the MA-XRF scanning has revealed one horizontal line and four quasi-vertical lines at the top of *PHerc.* 1021, ‘cornice’ 1 (Fig. 1A). Incidentally, the columns on cornice 1 do not represent a coherent fragment: the last column was originally a detached fragment^34^, but this is irrelevant for our conclusions. The horizontal line appears slightly undulated because of a deformation of the roll produced by the thermic shock linked to the eruption of Mount Vesuvius in AD 79, which caused the temperature to rise up to 320 °C^35,42^; the vertical ones are slightly slanting to the left in accordance with Maas’ Law. Comparison with the NIR image at 1000 nm of the same fragment, recorded during the same working sessions (Fig. 1B), shows that the horizontal line perfectly corresponds to the border between the upper margin of the roll and the top of the text columns. Furthermore, the four vertical lines coincide with the left border of each column.

The presence of another horizontal line, parallel to the revealed upper one, can be assumed at the bottom of the roll, marking the border between the lower margin and the bottom of the text; this part was not imaged by us because of time constraints. This is the simplest system of textual layout that we identified in the analyzed papyri: two parallel horizontal lines, for the upper and the lower margin, crossed by equally spaced parallel vertical lines, to signal the left border of each column. In this first system, vertical lines serve to mark only the intercolumnar space, i.e. the distance between the left border of each column and the left border of the next, without distinguishing between column and intercolumn within it.

### Delimitation of columns and intercolumns

Figures 2 and 3 report the results from *PHerc*. 1018, ‘cornice’ 1 and *PHerc*. 1420, ‘cornice’ 2. MA-XRF lead maps have revealed, at the top of *PHerc.* 1018, ‘cornice’ 1, one horizontal line and seven parallel quasi-vertical lines (Fig. 2A) and, in *PHerc.* 1420, ‘cornice’ 2, which was mapped in its entirety, again one horizontal line and seven parallel quasi-vertical lines (Fig. 3A). Again, direct comparison with the NIR images recorded on the same fragments by us (Figs. 2B and 3B) unambiguously correlate these lead-drawn lines with the text layout of the rolls.

The horizontal line corresponds to the border between the upper margin and the top of the text columns, while the vertical lines coincide with the left and the right borders of each column and each intercolumn. In *PHerc.* 1018, the last vertical line delimiting the last text column to the right is missing, and both vertical lines marking the adjacent intercolumn are no longer visible because of the papyrus deterioration in this area. Similarly, in *PHerc.* 1420, only the right part of the first column to the left and the left part of the last intercolumn to the right survive. The bottom area in *PHerc.* 1018 was not imaged by us due to time constraints, and that of *PHerc.* 1420 no longer survives. However, in both cases a second horizontal line, parallel to the upper one, should be assumed to mark the border between the lower margin and the bottom of the columns. Both papyri were also subject to heat damage (suffered during the volcanic eruption), so that, here too, the surviving horizontal line appears undulated (slightly in *PHerc.* 1018, more visibly in *PHerc.* 1420); in this case, too, the vertical lines are slightly slanting to the left, in accordance with Maas’ Law. *PHerc.* 1390/908 and *PHerc.* 558 also belong to the same layout system, as is shown in Figs. S3 and S4.

### Delimitation of columns, intercolumns and lines

Finally, we discuss the third and most elaborate layout system that we encountered, illustrated in Fig. 4. MA-XRF revealed, at the leftmost and the rightmost parts of *PHerc.* 164, ‘cornice 2’, ‘pezzo’ 1, two grids consisting of several horizontal and quasi-vertical lines (Fig. 4A and C). By comparing this map with the Infrared image at 950 nm of the same fragment produced by a team from Brigham Young University (Fig. 4B and D), the horizontal lines are found to exactly correspond to baselines used by the scribe as a guide for writing in straight lines inside the column, while the vertical lines coincide with the left and the right borders of each column and each intercolumn, as already encountered in the previous layout system; in this case, too, they are slightly slanting to the left, in accordance with Maas’ Law. The uppermost horizontal line delimits, as usual, the border between the upper margin and the top of each column of text. The number of horizontal lines recorded on these fragments is limited, since only a small part of the original bookroll is preserved, so only a few text lines per column survive. It should be assumed that, if the entire roll had been preserved in the original conditions, each ruling line would consistently correspond to a line of text.

## Discussion

The application of MA-XRF to Herculaneum papyri provides important experimental evidence that expands our knowledge of ancient Greek book production. Firstly, it confirms what we knew from classical authors, and was supposed by some modern scholars, about the use of a ruler and a leaden tool like a disk by scribes for the layout of ancient papyrus bookrolls. The sample analyzed by us is representative from a chronological, geographical and authorial point of view. Chronologically, the investigated rolls range from the full 2nd century BC (*PHerc.* 1420) to the end of the 1st century BC/beginning of the 1st century AD (*PHerc.* 164 and *PHerc.* 1018)^9^. Geographically speaking, while most of the samples have been produced between Rome and Campania, some of them probably stem from the Eastern Mediterranean, either Athens or Asia Minor or else Alexandria (*PHerc.* 1420 and *PHerc.* 1390/908)^9^. The authors represented are Epicurus, possibly Demetrius Laco and Philodemus. Different phases of formation of the Herculanean library are also included, from an earlier one documented by *PHerc.* 1420 to a post-Philodemean one represented by *PHerc.* 1018 and *PHerc.* 164^9^. This suggests that the layout of ancient Greek papyrus rolls described above was in all probability a common scribal practice in the most representative areas of the ancient Mediterranean from the middle of the Hellenistic age to the early imperial period. In particular, the simplest system of textual layout (see above: Results, *Delimitation of intercolumnar spaces*) seems typical of bookrolls of lower quality, which did not require any accurate *mise en page*. In this regard, it is no coincidence that the only investigated papyrus roll written with this layout, i.e. *PHerc.* 1691/1021, contains a graphically inaccurate and textually provisional redaction of Philodemus’ *Index Academicorum*, possibly the author’s own master copy^9,36^. The second layout system (see above: Results, *Delimitation of columns and intercolumns*) was found in bookrolls of intermediate or high quality, where a more careful distinction between written and blank spaces was demanded. Finally, the third layout system described above (see Results, *Delimitation of columns, intercolumns and lines*), being the most elaborate one, must have been associated with rolls of even higher quality, in agreement with our findings. It was in fact used for *PHerc.* 164, which holds the final calligraphic version of Philodemus’ *Index Academicorum*^9^.

A further conclusion inferable from this study concerns Maas’ Law, i.e. the tendency of the left border of each column to slant to the left (see above, *Introduction*), which is typical of both Greco-Egyptian^1,8^ and, although this has been a matter of controversy until recently, Herculaneum papyrus rolls^37,38^. Historically speaking, scholars have been divided between those who believe that this scribal phenomenon was unintentional and a sign of graphic inaccuracy^2^ and those who are convinced that it was, to the contrary, a deliberate operation undertaken with an aesthetic purpose^1^. The debate on this issue has remained open, up to now. The results arising from this research prove that Maas’ Law was indeed an intentional and both chronologically and geographically widespread scribal practice aimed at a more pleasant and more beautiful appearance of the bookroll. The observed presence of parallel quasi-vertical lines, i.e. lines that slightly slant to the left, drawn prior to the text redaction, supports this conclusion.

Curiously, as mentioned, the layout system by means of lead-drawn ruling lines detected by us in Herculaneum papyri and described above has so far not been found, or is not immediately visible, in Greco-Egyptian papyri. Nor has the application of advanced investigation techniques revealed, to the best of our knowledge, such a layout or any kind of lead-drawn ruling lines in these papyri. The few available studies of this kind are focused either on papyrus fragments of very limited size (a few centimeters) containing only text with no blank spaces^31^ or on documentary papyri, which—by definition—present characteristics totally different from those of literary papyri^24^. It is therefore recommendable to extend the application of MA-XRF to other literary papyri from Egypt, especially bookrolls in which both text columns and blank spaces are well preserved. The closest parallel to our case is *POxy*. LXXI 4809, which clearly exhibits the second layout system described above (Results, *Delimitation of columns and intercolumns*). However, in this case ruling lines are engraved, rather than drawn, with a sharpened instrument like a pin or a knife^39^. Vertical series of guide dots placed at the beginning, the middle or the end of each line, which recur either periodically or irregularly, were discovered by scholars in other Greco-Egyptian papyri^1,8^. Isolated dots at the beginning and/or the end of a line (usually the very first one) have also been detected in two Herculaneum papyri, i.e. *PHerc.* 1507 and *PHerc.* 1074a^38,40^. However, as has been remarked by scholars, these dots cannot have been served to trace ruling lines between which to write, i.e. as a way of lineation^8^. The relationship between these dots and the possible presence of lead-drawn ruling lines in ancient Greek papyri remains unclear for the simple reason that, as mentioned, MA-XRF has so far not been systematically applied to Greco-Egyptian papyri, nor was it applied to the two above-mentioned Herculaneum papyri possibly exhibiting guide dots. In conclusion, this study provides the first experimental evidence that in wide areas of the ancient Mediterranean from the middle of the Hellenistic age to the early imperial period Greek papyrus bookrolls were laid out through ruling lines, probably drawn by means of a ruler and a leaden disk, in agreement with classical literary sources and modern scholars’ suppositions. It also resolves the debate around Maas’ Law, proving that this was a deliberate scribal practice endowed with an aesthetic purpose.

## Methods

### Samples description

1. *PHerc.* 1691/1021 (second quarter of first century BC) was mechanically unrolled between 1782 and 1795 and contains a graphically inaccurate and textually provisional version of Philodemus of Gadara’s *Index Academicorum* (110-*post* 40 BC). It consists of nine papyrus fragments, contained in nine frames (‘cornici’), of which the fragment analyzed by us (*PHerc.* 1021, ‘cornice’ 1) measures 20.8 cm in height and 30.5 cm in length.
2. *PHerc.* 1018 (end of first century BC/beginning of first century AD) was unrolled in 1808 and holds Philodemus’ *Index Stoicorum*. It consists of twenty-one papyrus fragments stored in twelve ‘cornici’, of which the fragment analyzed by us (‘cornice’ 1) measures 17 cm in height and 25.6 cm in length.
3. *PHerc.* 1420 (second century BC) was unrolled in 1782 and contains, together with *PHerc.* 454 and *PHerc.* 1056, one of the three preserved copies of Book 25 of Epicurus’ masterpiece *On Nature*. It consists of six papyrus fragments gathered in two ‘cornici’, of which the fragment analyzed by us (‘cornice’ 2) measures 13.1 cm in height and 24 cm in length.
4. *PHerc.* 1390/908 (second century BC) was unrolled between 1804 and 1805 and contains a work *On human procreation* recently attributed to the Epicurean philosopher Demetrius Laco^41^. It consists of seven papyrus fragments collected in five ‘cornici’, of which the fragment analyzed by us (‘cornice’ 4) measures 12.9 cm in height and 30.1 cm in length.
5. *PHerc.* 558 (third quarter of first century BC) was unrolled in 1888 and contains the so-called *History of Socrates and the Socratics* by Philodemus. It consists of twenty-seven papyrus fragments contained in four ‘cornici’, of which the fragment analyzed by us (contained in ‘cornice’ 3) measures 9.3 cm in height and 8.5 cm in length.
6. *PHerc.* 164 (end of first century BC/beginning of first century AD) was unrolled in 1805 and contains the final calligraphic version of Philodemus’ *Index Academicorum*. It consists of eleven papyrus fragments contained in three ‘cornici’, of which the fragment analyzed by us (contained in ‘cornice’ 2) measures 6.4 cm in height and 37.1 cm in length.

The thickness of the papyrus sheet is about 0.15 mm. Each fragment is glued to a paperboard, which is slightly thicker than the papyrus sheet (ca. 0.30 mm), and is itself fastened to a piece of cardboard. All fragments are distributed across metallic frames (“cornici”), which are kept in the Officina dei Papiri Ercolanesi of the National Library “Vittorio Emanuele III” of Naples.

### MA-XRF imaging set-up

The MA-XRF imaging investigation of the papyrus fragments was carried out using a novel mobile MA-XRF scanner developed at the XRAYLab of CNR-ISPC Catania (Italy). The new system is based on a real-time technology which was previously developed by some of the Authors^15^. However, it recently underwent a major upgrade, conveyed by the development of new high-performing mechatronics and a high-throughput detection system. The main component of the device is the spectrometric head, which consists of an X-ray source and a fluorescence detection system. Samples are irradiated with a Rh anode microfocus source, equipped with a high gain polycapillary with a focus of 50 μm at 15 mm distance, measured at 10 keV radiation. The fluorescence radiation induced by the primary beam is detected with a hodoscope detector composed of 6 SDDs operated in parallel and arranged in an 3D annular compact geometry (Fig. S1). Each SDD detector is collimated to 40 mm^2^ with a resulting total active area of 240mm^2^. The measurement geometry is 90°–45°, with the possibility to slightly adjust the detection angles to orient the SDDs on the irradiated spot at the sample surface when operations are performed out of focus. Sample to detectors distance is 19 mm at the focus. A figure of merit of the spectrometric capabilities of the hodoscope is given in Fig. S2. The measurement head is installed on a mechatronic device composed of 3 linear axes (XYZ) and a real-time processing unit (CPU) with high computational capabilities. The scanning can be performed either with samples positioned in vertical (i.e., XY directions with focal distance along Z) or horizontal (i.e., XZ directions with focal distance along Y). The scanning area in the vertical configuration is 120 × 90 cm^2^. In this case, the Z axis with a 20 cm travel-range is used for the initial alignment procedures and for the dynamical correction of the measurement distance during the scanning. This correction is operated in sync with a laser distance sensor through the CPU. The horizontal configuration is used for fragile samples that need to be kept on a table-top, as is the case of the papyrus fragments analyzed in the present work.

The acquisition of XRF pixel spectra is performed in continuous, using a fast DXP working in mapping mode. During the measurements, a series of TTL signals is given by the CPU to synchronize the axis position along the scanning direction and the XRF pixel acquisition. The minimum dwell-time for 1 mm pixel size is 7 ms corresponding to a maximum scanning speed of about 150 mm/sec. The CPU is developed in a multi-node design with high-computing capabilities distributed among an FGPA, a XEON multi-core server and two ancillary 9th generation Intel multi-core processors. It is programmed in a real-time environment and manages sensors and scanning stages, I/O gates, data acquisition, and data processing. All measurement parameters are entered and monitored by the users through an interactive dashboard. Finally, the use of a custom-programmed software allows the users to obtain on the fly the deconvoluted elemental distribution images of the samples and to apply, during the scanning, several analytical tools for augmenting the data elaboration and interpretation.

### Analysis of Herculaneum Papyri

The MA-XRF measurements of the Herculaneum papyri were performed in the horizontal configuration with typical pixel-size and dwell-time of 250 μm and 10 ms respectively (Fig. S5). To better bring out the ruling lines detected from the on-the-fly analysis, some samples were additionally measured in smaller regions with a higher lateral resolution of 100 μm, and 50 ms dwell time. For each mapped fragment, the elemental composition is investigated by integrating the fluorescence radiation across the full map to obtain the sum spectrum (Fig. S6A), from which the major chemical elements are easily identified despite the high background in the spectrum, due to the scattered radiation. To facilitate the identification of trace elements, the maximum pixel spectrum is derived, by showing for each energy the highest recorded intensity (Fig. S6B). In this fashion, the XRF background is not integrated, and characteristic emission lines are enhanced, even for chemical elements only present in a small area of the papyrus roll. Finally, the evaluation of the spatial distribution of the identified chemical elements is obtained by performing on the fly the deconvolution of the XRF pixel spectra, to extract the net intensity of each emission line in relation with the pixel coordinates (Fig. S7). Regarding the lead, its distribution was mapped through both M-lines and L-lines. The latter show a strong aggregation of Pb along the border of gaps in the samples investigated. The effect is probably due to external contamination caused by the eruption of Mount Vesuvius in AD 79 or to migration phenomena of lead in the papyrus support due to thermal shock. This last aspect deserves further investigation, which is beyond the scope of the present study. However, the lead aggregation in the gaps extends to the full thickness of the samples and affects the quality of the visualization of the weak signal coming from the Pb traces in the ruling lines at the surface. For this reason, we have used the Pb-M images to visualize the results in Figs. 1, 2, 3, 4, S3, and S4 where the detected Pb-M signal come from the surface for both the ruling lines and the aggregated lead in the gaps. A comparison of Pb-L and Pb-M images is given for *PHerc.* 1018, ‘cornice’ 1, in Fig. S9.

**Figure 5:**
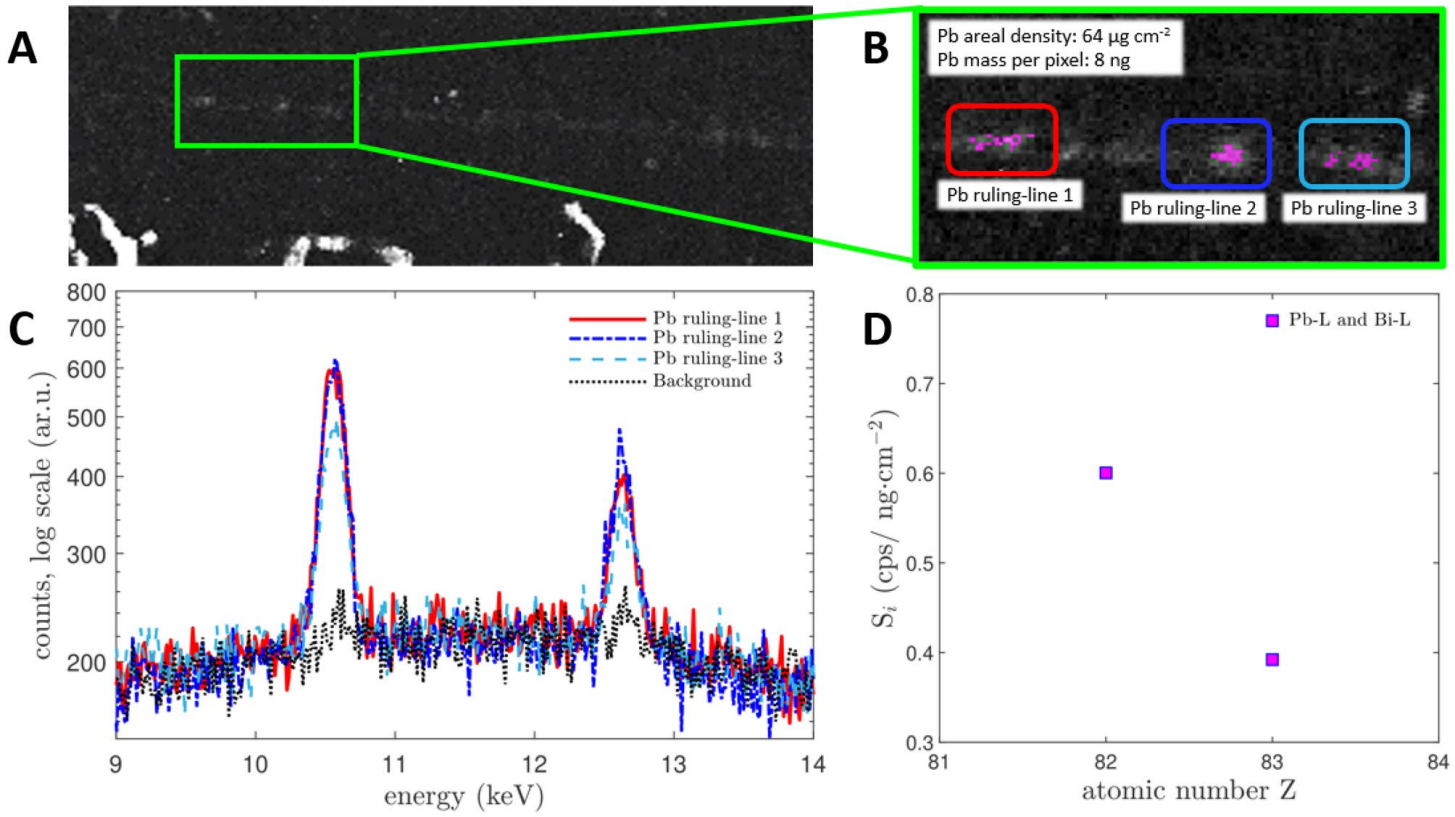
Lead estimation procedure. Pb-Lα map of *PHerc*. 1018, ‘cornice’ 1 taken for this sample at a scanning step size of 100 μm and dwell time of 50 ms (**A**), zoomed area, showing the sampled selection, purple pixels, within the labelled color-coded regions (**B**), Pb-L signal in the integrated spectra of the selected pixels along the ruling line and on the background obtained by randomly sampling pixels across the papyrus rolls, outside of the ruling lines, in logarithmic scale (**C**) and Sensitivity (S_i_) of the MA-XRF scanner in the energy region of the Pb-L lines (**D**).

Semi-quantitative information on the residual Pb content, along the ruling-lines of the papyrus rolls, were extracted using the sensitivity for lead, obtained for our MA-XRF set-up by measuring thin-target reference materials. The quantification procedure for the papyrus rolls is illustrated in Fig. 5, for the case of *PHerc.* 1018, ‘cornice’ 1. From the Pb-Lα intensity maps, regions of interest were identified along the Pb ruling-lines. Along the ruling lines, the pixels with higher relative intensity were hand-picked for the analysis. For comparisons, region of interest with the same number of pixels were randomly selected in the papyrus background. The extracted fluorescence curves were then modelled to evaluate the Pb-L net counts. The results of this analysis are summarized in Tab. S1, and represent upper limits for the Pb areal density. Also, the absolute mass of lead observed pixel by pixel was evaluated, to an amount of ~ 8 ng per pixel along the ruling-lines (upper limit) and ~ 1 ng per pixel in the areas outside of the Pb ruling-lines. This estimate was derived assuming the typical pixel size of 250 μm, and keeping into account the beamspot diameter of 50 μm.

## Supplementary Information

Supplementary Information.

## Data Availability

All data needed to evaluate the conclusions in the paper are present in the paper and/or the Supplementary Information. Additional data available from authors upon request.
