# Supplementary material for: Layout of ancient Greek papyri through lead-drawn ruling lines revealed by Macro X-Ray Fluorescence Imaging
Source: Sci Rep. 2023 Apr 21;13:6582. doi: 10.1038/s41598-023-33242-8 (PMC10121553; doi:10.1038/s41598-023-33242-8)
Supplement: Supplementary file 1 — Supplementary Information. [file 41598_2023_33242_MOESM1_ESM.pdf]

Supplementary Information for

Layout of ancient Greek papyri through lead-drawn ruling lines revealed by Macro X-ray Fluorescence Imaging

Francesco P. Romano, Enzo Puglia, Claudia Caliri, Danilo P. Pavone, Michele Alessandrelli, Andrea Busacca, Claudia G. Fatuzzo, Kilian J. Fleischer, Carlo Pernigotti, Zdenek Preisler, Christian Vassallo, Gertjan Verhasselt, Costanza Miliani*, Graziano Ranocchia*

*Corresponding authors: Costanza Miliani, Graziano Ranocchia

Email: costanza.miliani@cnr.it; graziano.ranocchia@unipi.it

This PDF file includes:

NIR technical photography
Fig. S1 – Hodoscope detector installed on the MA-XRF set-up
Fig. S2 – Sensitivity (S_i) and Limits of Detection (LoD_i) of the new MA-XRF set-up
Fig. S3 – *PHerc.* 1390/908, ‘cornice’ 4
Fig. S4 – *PHerc.* 558, ‘cornice’ 3, ‘pezzo’ 7
Fig. S5 – The MA-XRF scanner during the investigation of Herculaneum papyri
Fig. S6 – MA-XRF integrated spectrum and maximum-pixel spectrum of *PHerc.* 1420, ‘cornice’ 2
Fig. S7 – MA-XRF elemental distribution images of *PHerc.* 1420, ‘cornice’ 2
Tab. S1 – Lead semi-quantitative results along and outside the ruling lines
Fig. S8 – *PHerc.* 1021, cornice 1
Fig. S9 – Pb-L and Pb-M distribution images of *PHerc.* 1018, ‘cornice’ 1

NIR technical photography

The infrared pictures of the papyri were recorded with a modified full-spectrum camera (Nikon D800E) equipped with 50mm and F1:1.8 optic (AF-Nikkor-D) and a 1000nm IR filter (Edmund Optics). The camera was mounted on a tripod at a 65cm distance to the shooting plane. The diaphragm was operated at F5.6, with a near focus distance of 63cm and a far focus distance of 68cm. To maintain uniformity of the magnification ratio across images of different 'cornici' of the same papyrus fragment, all the frames were recorded with the same focal point and camera to subject distance. The manual focusing was calibrated to the infrared wavelength and, to achieve a higher level of control, a greyscale and a metric ruler were positioned on the shooting plane. The light was provided by two 150 W incandescent (tungsten filament) lamps, placed one to the right and one to the left of the shooting set, with distance to the subject of roughly 150cm. The images were shot remotely from a laptop with the aid of the live view instrument, to verify both the focusing and the histogram of exposure. The photographic frames, which were taken in 14 bit RAW format, were processed with the Capture One Pro software and then exported in the flexible 8 bit TIFF format.

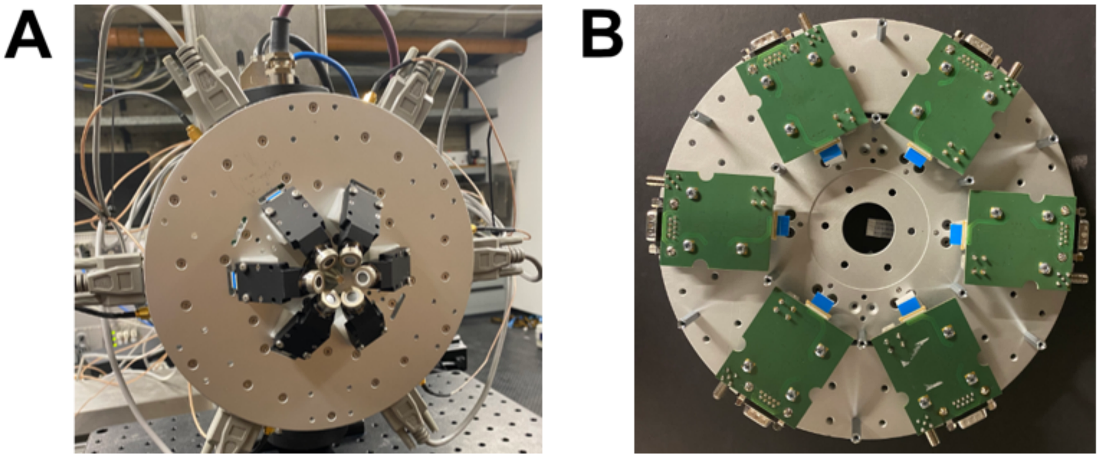

Fig. S1. Hodoscope detector: front view of the 3D annular assembly of 6SDDs (A) and image of the back side of the round custom support, showing the integrated electronics of each detector (B). The 6 SDDs are operated in parallel, in 90deg-45deg measurement geometry with sample to detectors distance of 19mm. It is possible to slightly adjust the detection angles to perform measurements with the irradiated spot out of the beam focus, with a different sample to detector distance.

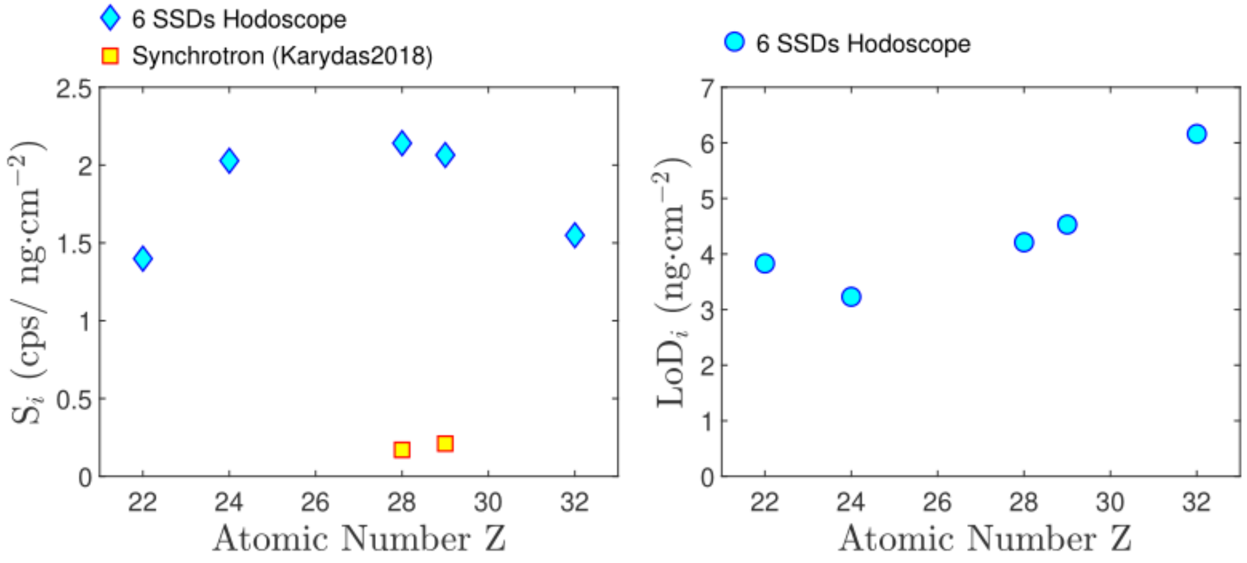

Fig. S2. Sensitivity (S_i) and Limits of Detection (LoD_i) of the new MA-XRF set-up, compared with literature data from the XRF beamline at ELETTRA synchrotron [32]. The values were extracted from Micromatter™ reference single metal evaporation samples, measured for 300 sec with Rh tube settings of 50kV and 600μA. S_i values are evaluated using the K lines net counts, while the LOD_i are estimated by evaluating the net counts and underlying background of the Kα lines. The collection of the fluorescence radiation by six independent detectors operated in parallel, each with its own amplifier, allows a six-fold increase of the detection solid angle while the acquisition time and dead time remain unchanged, with respect to the use of a single detector. The extracted sensitivity for Ni and Cu is in the order of 2 cps per ng·cm^{-2}, corresponding to an improvement of an order of magnitude when compared with that of a XRF synchrotron set-up [32]. For lead, we extracted S_{Pb}=0.6 cps/ng·cm^{-2} (using the L group net counts) and LOD_{Pb}=1.4 ng·cm^{-2} (Lα line).

A

B

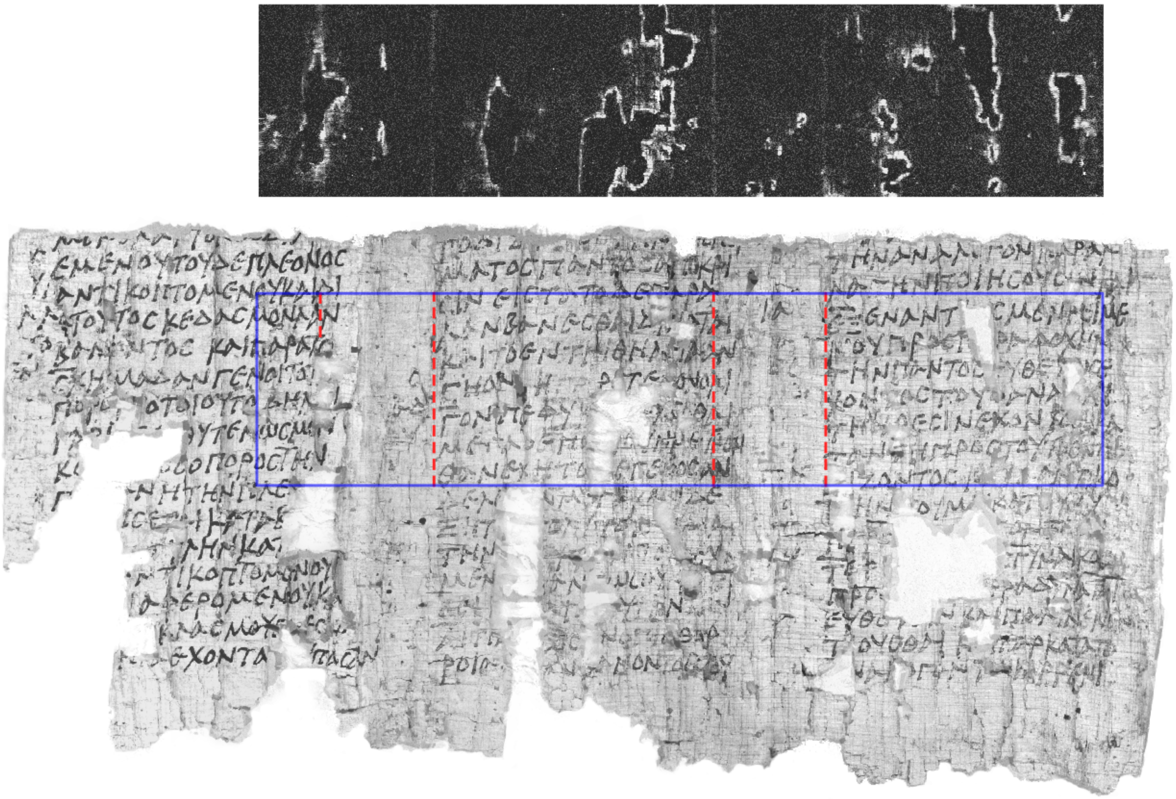

Fig. S3. ***PHerc.* 1390/908, ‘cornice’ 4.** Pb-M distribution map obtained through MA-XRF Imaging (scanning area 22 × 5 cm^2). By permission of the Ministero della Cultura (photo credit: Biblioteca Nazionale “Vittorio Emanuele III,” Napoli—Consiglio Nazionale delle Ricerche, Istituto di Scienze del Patrimonio Culturale) (**A**) and NIR image at 950 nm. Red lines mark the borders of each column and intercolumn; the blue rectangle marks the sample area imaged by us. By permission of the Ministero della Cultura (photo credit: Biblioteca Nazionale “Vittorio Emanuele III,” Napoli—Brigham Young University, Provo) (**B**).

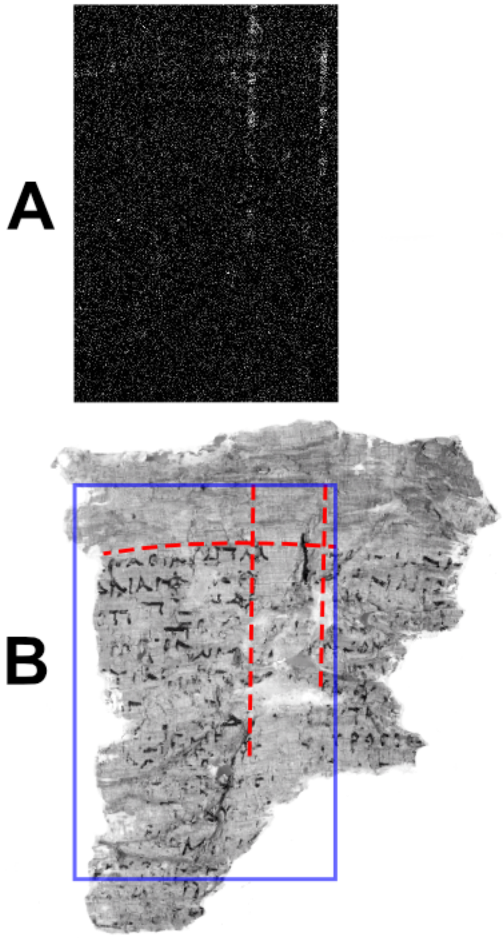

Fig. S4. ***PHerc.* 558, 'cornice' 3, 'pezzo' 7.** Pb-M distribution map obtained through MA-XRF Imaging (scanning area 5 × 7.5 cm^2). By permission of the Ministero della Cultura (photo credit: Biblioteca Nazionale "Vittorio Emanuele III," Napoli—Consiglio Nazionale delle Ricerche, Istituto di Scienze del Patrimonio Culturale) (**A**) and NIR image at 1000 nm. Red lines mark the borders of each column and intercolumn; the blue rectangle marks the sample area imaged by us. By permission of the Ministero della Cultura (photo credit: Biblioteca Nazionale "Vittorio Emanuele III," Napoli—Consiglio Nazionale delle Ricerche, Istituto di Scienze del Patrimonio Culturale) (**B**).

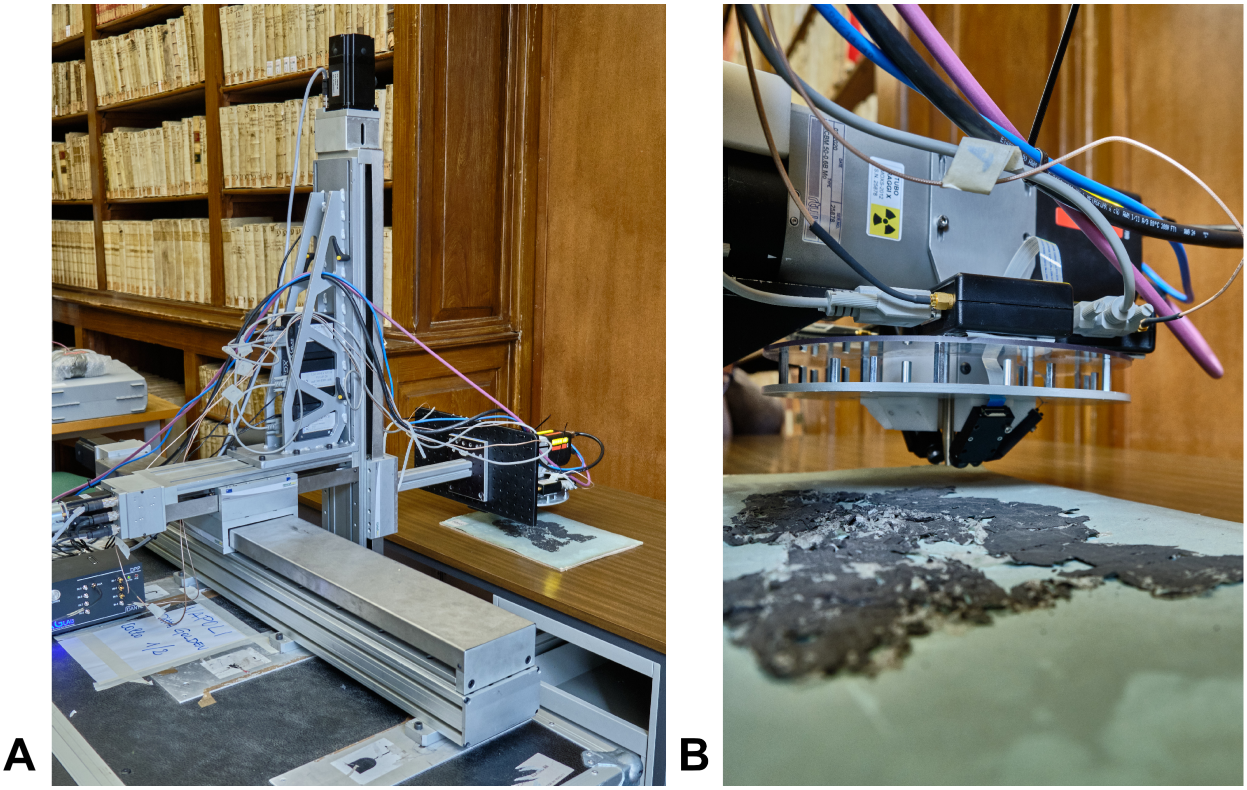

Fig. S5. The MA-XRF scanner during the investigation of Herculaneum papyri: general view (A) and detail (B). The instrument can be operated for either vertical or horizontal scanning. This latter configuration was used for the measurements of Herculaneum papyri. The samples were measured with a continuous scanning at a speed of 2.5mm/s and a lateral resolution of 250µm per pixel. The corresponding dwell-time is 10ms. The X-ray source was operated at 50kV and 600µA.

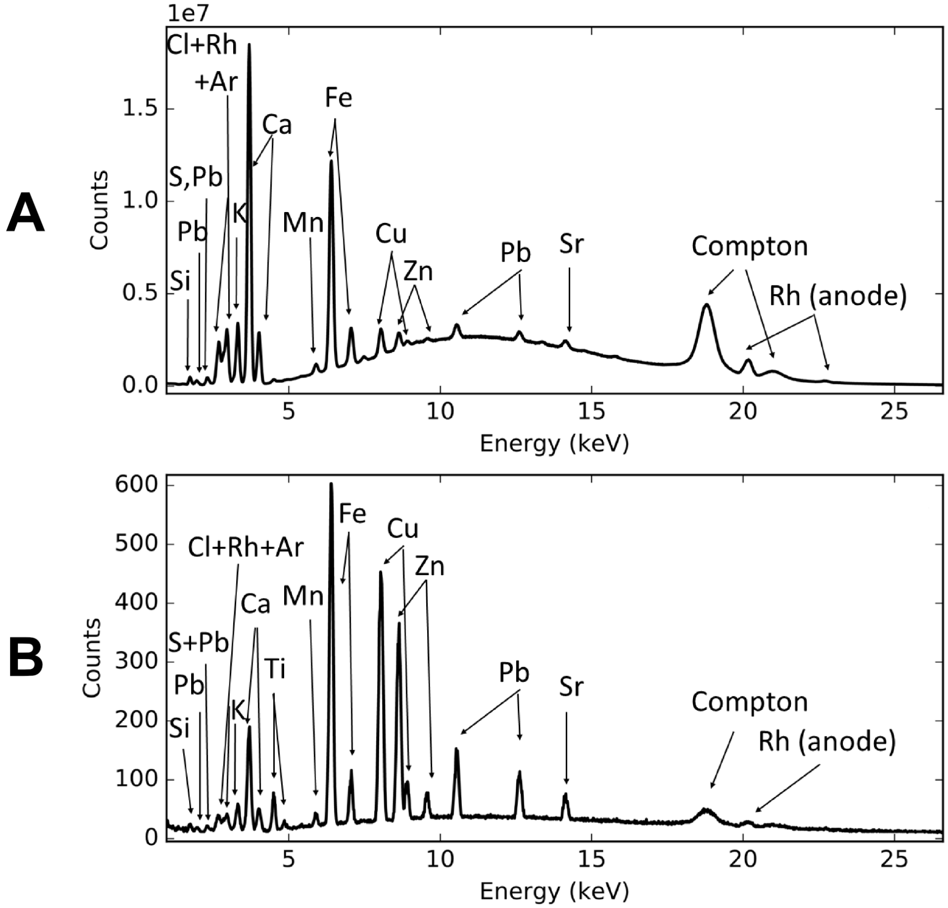

Fig. S6. MA-XRF integrated spectrum recorded over the entire analyzed area of papyrus fragment *PHerc.* 1420, 'cornice' 2 (A) and the corresponding MA-XRF maximum pixel spectrum (B). The full scanning of the sample contains 667000 pixels. In these spectra are evident the relevant chemical elements characterizing the fragment, namely chlorine (Cl), potassium (K), calcium (Ca), iron (Fe), manganese (Mn), strontium (Sr) and lead (Pb). Owing to the high background in the integrated spectrum, signals coming from spot-aggregations of a specific element or from trace elements are not well detected. These latter (i.e., Ti, Cu, Zn and Pb) are better identified in the maximum pixel spectrum, where the maximum recorded intensity for each fluorescence energy is shown.

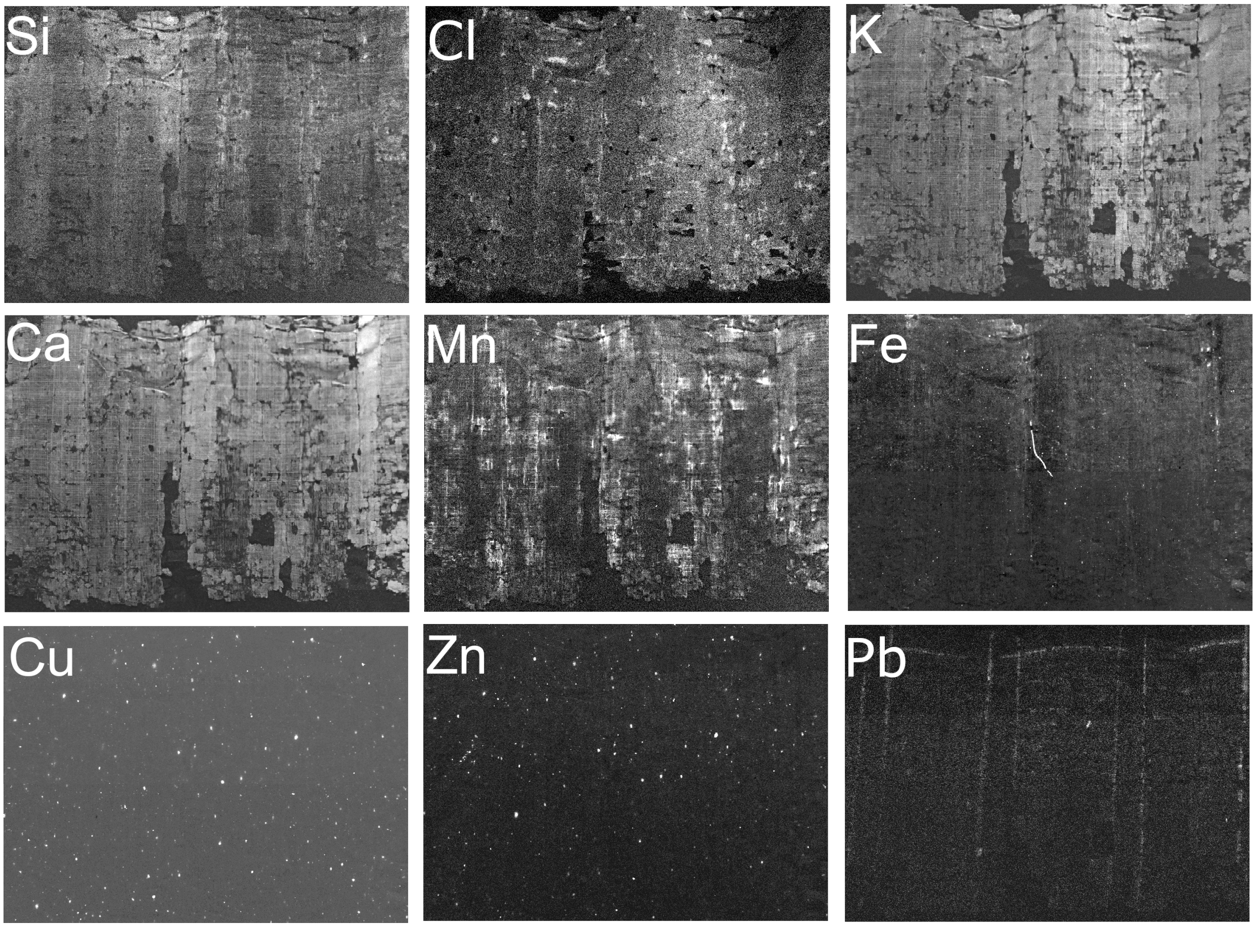

Fig. S7. MA-XRF elemental distribution images of papyrus fragment *PHerc.* 1420, 'cornice' 2. The distributions of calcium and potassium well highlight the gaps and the texture of the fragment, emphasizing the area where thickness is larger. Most of the elements in the maps (i.e., iron, manganese, copper, titanium, zinc, and silicon) could be associated with environmental contaminations or soil deposit. Finally, the distribution of lead reveals the layout of the text columns as discussed in the work.

Papyrus	Location	Pb areal density ($\mu g \cdot cm^{-2}$)	Pb per pixel (ng)
PHerc. 1018	Pb ruling-line 1	63	7.9
	Pb ruling-line 2	66	8.2
	Pb ruling-line 3	45	5.7
	background	6	0.8
PHerc. 164	Pb ruling-line 1	67	8.4
	Pb ruling-line 2	68	8.4
	Pb ruling-line 3	74	9.2
	background	8	1
**Average value**	**Pb on the ruling lines**	**64**	**8.0**
	**Pb on the background**	**7**	**0.9**

Table S1. Lead semi-quantitative results along and outside the ruling lines. The recorded intensity was integrated to reach 1sec of acquisition time. In the last column, the areal density was normalized to the single pixel area, to show the quantity of Pb which emits fluorescence pixel by pixel. For direct comparison, the pixel area was assumed to be a rectangle of sides 250μm (typical sampling size) and 50μm (beam spot diameter) for all scans. The error on the estimates, mainly due to the statistical uncertainty, is in the order of 10% along the Pb ruling lines and 30% on the background. The extracted values represent upper limits for the lead content.

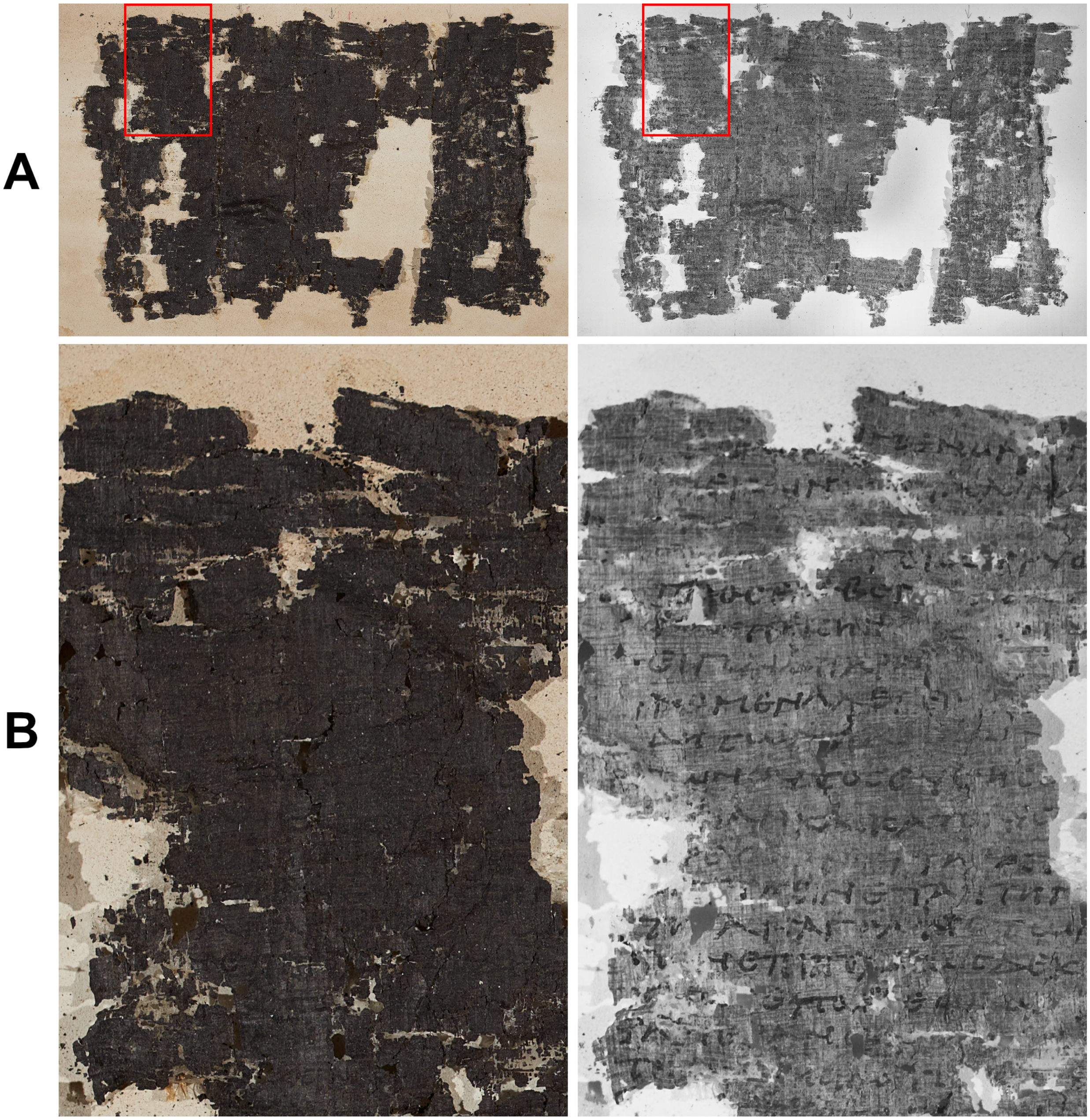

Fig. S8. ***PHerc.* 1021, cornice 1.** VIS and NIR photographies. By permission of the Ministero della Cultura (photo credit: Biblioteca Nazionale “Vittorio Emanuele III,” Napoli—Consiglio Nazionale delle Ricerche, Istituto di Scienze del Patrimonio Culturale) **(A)** and a detail of the same fragment displaying Greek text barely guessable in the visible but clearly distinguishable in the corresponding NIR image, taken with a 1000nm filter. By permission of the Ministero della Cultura (photo credit: Biblioteca Nazionale “Vittorio Emanuele III,” Napoli—Consiglio Nazionale delle Ricerche, Istituto di Scienze del Patrimonio Culturale) **(B).**

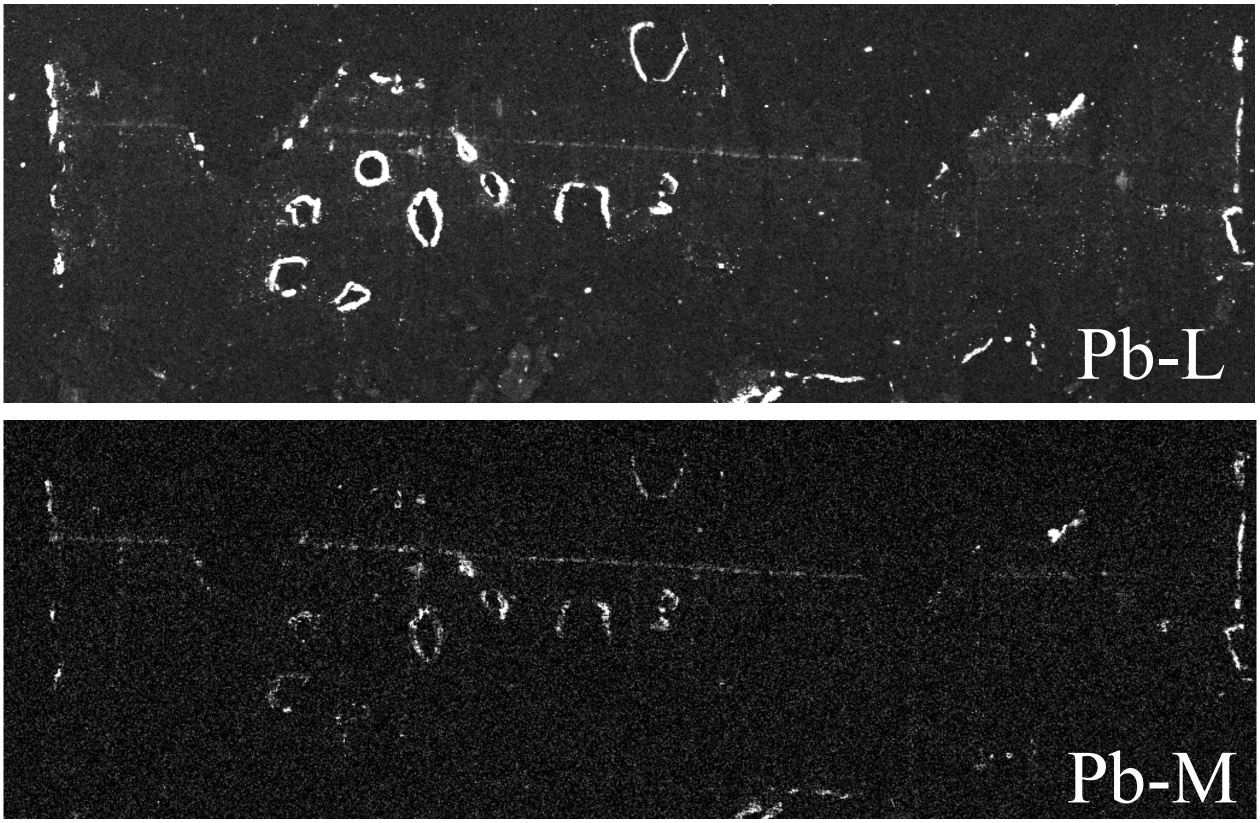

Fig. S9. Pb-L and Pb-M distribution images of papyrus fragment *PHerc.* 1018, 'cornice' 1. Pb-L distribution image evidences a strong aggregation of lead along the border of the gaps presumibly originated during the volcanic eruption. This affects the visualization of the Pb ruling lines that are still evident but with less detail with respect to that of the Pb-M map. By permission of the Ministero della Cultura (photo credit: Biblioteca Nazionale "Vittorio Emanuele III," Napoli—Consiglio Nazionale delle Ricerche, Istituto di Scienze del Patrimonio Culturale)
